# Severe impact of late diagnosis of congenital adrenal hyperplasia on gender identity, sexual orientation and function: case report and review of the literature

**DOI:** 10.3389/fgene.2022.902844

**Published:** 2022-10-31

**Authors:** Chiara Simeoli, Cristina de Angelis, Alessandra Delli Veneri, Davide Menafra, Nicola Di Paola, Claudia Pivonello, Carolina Di Somma, Paolo Valerio, Daniela Melis, Carlo Alviggi, Annamaria Colao, Rosario Pivonello

**Affiliations:** ^1^ Dipartimento di Medicina Clinica e Chirurgia, Sezione di Endocrinologia, Università Federico II di Napoli, Naples, Italy; ^2^ Dipartimento di Medicina Clinica e Chirurgia, Sezione di Endocrinologia, Unità di Andrologia e Medicina della Riproduzione e della Sessualità Maschile e Femminile (FERTISEXCARES), Università Federico II di Napoli, Naples, Italy; ^3^ Dipartimento di Neuroscienze, Anestesiologia e Farmacologia, Unità di Psicologia Clinica, Università Federico II di Napoli, Naples, Italy; ^4^ Dipartimento di Sanità Pubblica, Università Federico II di Napoli, Naples, Italy; ^5^ Dipartimento di Medicina, Chirurgia e Odontoiatria, Sezione di Pediatria, “Scuola Medica Salernitana”, Salerno, Italy; ^6^ Department of Neuroscience, Reproductive Science and Odontostomatology, University Federico II, Naples, Italy; ^7^ Unesco Chair for Health Education and Sustainable Development, University Federico II, Naples, Italy

**Keywords:** Congenital adrenal hyperplasia, disorders of sex development, genital ambiguity, gender identity, gender dysphoria, sexual orientation, sexual dysfunction

## Abstract

Congenital adrenal hyperplasia (CAH) due to 21-hydroxylase deficiency (21-OHD) represents the most frequent form of CAH and of 46, XX disorder of sex development in female newborns. In the majority of cases, particularly in developed countries, female patients suffering from the classic forms of CAH reach the diagnosis at birth or in the early childhood, allowing a prompt treatment with a correct gender assignment. The current manuscript describes an unusual case of an Italian 46-year-old woman, homeborn in the 60s, receiving an extraordinarily late diagnosis of simple virilising classic form of CAH due to 21-OHD, determining a relevant impairment of both physical and psychosexual development. The patient presented primary amenorrhea, height under target, overweight with visceral adiposity, hypercholesterolemia and insulin resistance, hirsutism with a typical male-pattern hair growth, external genital ambiguity, and a severe impairment in the entire series of psychological dimensions, particularly severe depressive symptoms, together with gender dysphoria relative to the female gender assigned at birth, cross-gender behaviours, and body image discomfort, which were associated with homosexual orientation, and sexual dysfunction. Following diagnosis and glucocorticoid (GC) replacement therapy, the hyperandrogenism control and familial and socio-cultural factors changes, particularly, living alone and the interruption of social isolation, were accompanied by menarche appearance, improvement in hirsutism and metabolic profile, and a resolution in all psychological dimensions, depressive symptoms, and gender dysphoria. The patient began to perceive homosexual orientation without discomfort, and ameliorating sexual function. Few cases of female patients with CAH due to 21-OHD receiving an extremely delayed diagnosis have been published. However, to the best of our knowledge, this is the first case including a complete psychosexual assessment at diagnosis with a detailed re-evaluation after 5 years of disease treatment.

## Introduction

Congenital adrenal hyperplasia (CAH) due to 21-hydroxylase (21-OH) deficiency (21-OHD) is the most common form of CAH, since it accounts for at least 95% and up to 99% of cases, and the most common form of 46, XX disorder of sex development (DSD) (Clayton et al., 2002; Murphy et al., 2011; Gidlöf et al., 2013; Lee et al., 2016; Speiser et al., 2018; Claahsen-van der Grinten et al., 2022). CAH due to 21-OHD is an autosomal recessive disorder characterized by an impairment of cortisol and, eventually, aldosterone synthesis, associated with an increase of adrenocorticotropic hormone (ACTH) secretion, with consequent adrenal hyperplasia and androgen excess (Speiser et al., 2018). The clinical phenotype is classified as classic or non-classic, depending on disease features and severity (Speiser et al., 2018). The classic form, characterized by neonatal or childhood onset, includes the salt wasting form, with severe cortisol and aldosterone insufficiency, and the simple virilising form, with mild to moderate cortisol and absent to mild aldosterone insufficiency (Speiser et al., 2018). The clinical picture of the classic form is enriched by external genital ambiguity exclusively in the female patients; indeed, prenatal exposure to androgen excess exerts a virilising effect, leading to changes in external genitalia, ranging from isolated clitoromegaly to severe clitoral enlargement acquiring a phallus appearance, with the internal female genital organs, including ovaries, fallopian tubes and uterus, remaining normal according to a female sex (Murphy et al., 2011; Gidlöf et al., 2013; Lee et al., 2016). However, the external genital ambiguity in the female foetus is likely not the unique consequence of prenatal exposure to androgen excess in the classic form, since in this condition prenatal androgen excess has been suggested to lead to masculinization of the brain with consequent masculinization of gender identity and/or role, with a prevalent non-heterosexual orientation being associated with gender and role modification or even isolated manifestation (Kuhnle et al., 1995; Zucker et al., 1996; Hines et al., 2004; Dessens et al., 2005; Meyer-Bahlburg et al., 2006a; Meyer-Bahlburg et al., 2008; Frisén et al., 2009; Hines et al., 2015; Meyer-Bahlburg et al., 2016; Razzaghy-Azar et al., 2017; Spencer et al., 2017; Daae et al., 2020). Indeed, several studies on female patients with classic forms of CAH due to 21-OHD reported a higher frequency of gender-variant identity development (Meyer-Bahlburg et al., 2006a; Razzaghy-Azar et al., 2017) and gender dysphoria (Dessens et al., 2005), cross-gender role behaviours (Spencer et al., 2017) and choice of male-dominant occupations (Frisén et al., 2009), non-heterosexual fantasies and sexual relationships (Zucker et al., 1996; Meyer-Bahlburg et al., 2008; Frisén et al., 2009; Daae et al., 2020), compared with either non-affected relatives or healthy subjects. Moreover, mainly in the most severe classic forms, sexual dysfunction, especially dyspareunia and impairment of sexual satisfaction, have been frequently observed (Gastaud et al., 2007; Frisén et al., 2009; Schernthaner-Reiter et al., 2019).

Nowadays, in the majority of cases, particularly in developed countries, the diagnosis of the classic form of CAH due to 21-OHD in female patients is reached at a prenatal, neonatal or early childhood stage, allowing a correct gender assignment and a prompt treatment with adequate doses of glucocorticoids (GCs), which are needed to replace cortisol and aldosterone deficiency and to control androgen excess (Speiser et al., 2018); medical treatment optimization and monitoring are dynamic processes, aimed at obtaining the challenging balance between under-treatment and over-treatment (Clayton et al., 2002; Speiser et al., 2018; Claahsen-van der Grinten et al., 2022).

The current manuscript describes a case of a woman, homeborn in Italy during the 60s with the support of a midwife, with an assigned female sex at birth despite ambiguous external genitalia, raised as a female but with a self-perception of a male during the entire life, and receiving an extraordinarily late diagnosis of simple virilising classic form of CAH due to 21-OHD during middle age. This case will evidence the relevant deleterious impact of the delayed diagnosis on the patient’s physical aspect and psychosexual development. To the best of our knowledge, this is one of the few cases of female patients suffering from classic form of CAH due to 21-OHD, who received an extremely delayed diagnosis, with the unique novelty of a complete psychosexual assessment at diagnosis and a detailed description of psychosexual changes following treatment during a long-term follow-up period.

## Case description

A 46-year-old Italian woman was admitted to the Endocrinology, Andrology and Metabolism Unit of ‘‘Federico II’’ University of Naples for the evaluation of a unilateral adrenal tumour (51 mm × 31 mm), incidentally discovered at ultrasound performed for abdominal pain.

### Baseline clinical evaluation

The clinical interview and the clinical evaluation revealed primary amenorrhea, height under target [158 cm; Standard Deviation Score −0.9; target height: 164 cm], overweight with visceral adiposity, skin hyperpigmentation, and hirsutism with a typical male-pattern hair growth. The biochemical assessment revealed hypercholesterolemia and insulin resistance. Genital examination showed ambiguous genitalia with a “phallus” of 7 cm and a vaginal ostium surrounded by hypoplastic minor labia and hypertrophic major labia; a careful palpation of inguinal regions failed to identify formations compatible with male gonads. This adult genital phenotype appeared to testify that a partial labio-scrotal fusion together with the enlargement of the clitoris, with ensuing virilization of female external genitalia, occurred during sexual differentiation, due to prenatal androgen excess. Based on clinical findings, the first diagnostic hypothesis was CAH. The hormonal assessment demonstrated low-normal cortisol, normal renin and aldosterone, elevated ACTH, testosterone, androstenedione and, particularly, 17-hydroxyprogesterone (17-OHP) levels, which confirmed the suspicion of CAH, probably due to 21-OHD. An ACTH stimulation test at standard dose (250 µg) confirmed the presence of adrenal insufficiency (serum cortisol t0’: 81.2 ng/ml, t60’: 92.2 ng/ml, normal ranges t60’ >200 ng/ml) and confirmed the diagnosis of CAH due to 21-OHD (serum 17-OHP t0’: 510 ng/ml, t60’: 842 ng/ml). On the basis of the absence of a relevant clinical syndrome suggestive of adrenal insufficiency and of the evidence of low-normal basal cortisol levels, a diagnosis of a long-term subtle adrenal insufficiency was performed. Magnetic resonance imaging, performed to better characterize the unilateral adrenal tumour incidentally discovered at ultrasound, and to evaluate the presence of internal female genital organs, revealed bilateral adrenal hyperplasia, without evidence of the adrenal tumour incidentally discovered at ultrasound, together with bilateral gonads and Müllerian structures (fallopian tubes, uterus and cervix, and proximal vagina); the imaging features of the bilateral gonads were more consistent with ovaries than testes. Chromosomal analysis, performed to exclude different concomitant chromosomal disorders and to confirm the female sex, revealed a 46, XX karyotype. The molecular analysis identified two 21-OH gene mutations (P30L and R356W compound heterozygosity). On the basis of clinical, biochemical, hormonal, imaging and genetic evaluations, a diagnosis of simple virilising classic form of CAH due to 21-OHD was confirmed. Glucocorticoid (GC) treatment was started using prednisone at an initial dose of 7.5 mg/day, gradually titrated up to 15 mg/day during the following 12 months, in order to primarily and rapidly control androgen excess, and secondarily replace the subtle cortisol deficiency.

**FIGURE 1:**
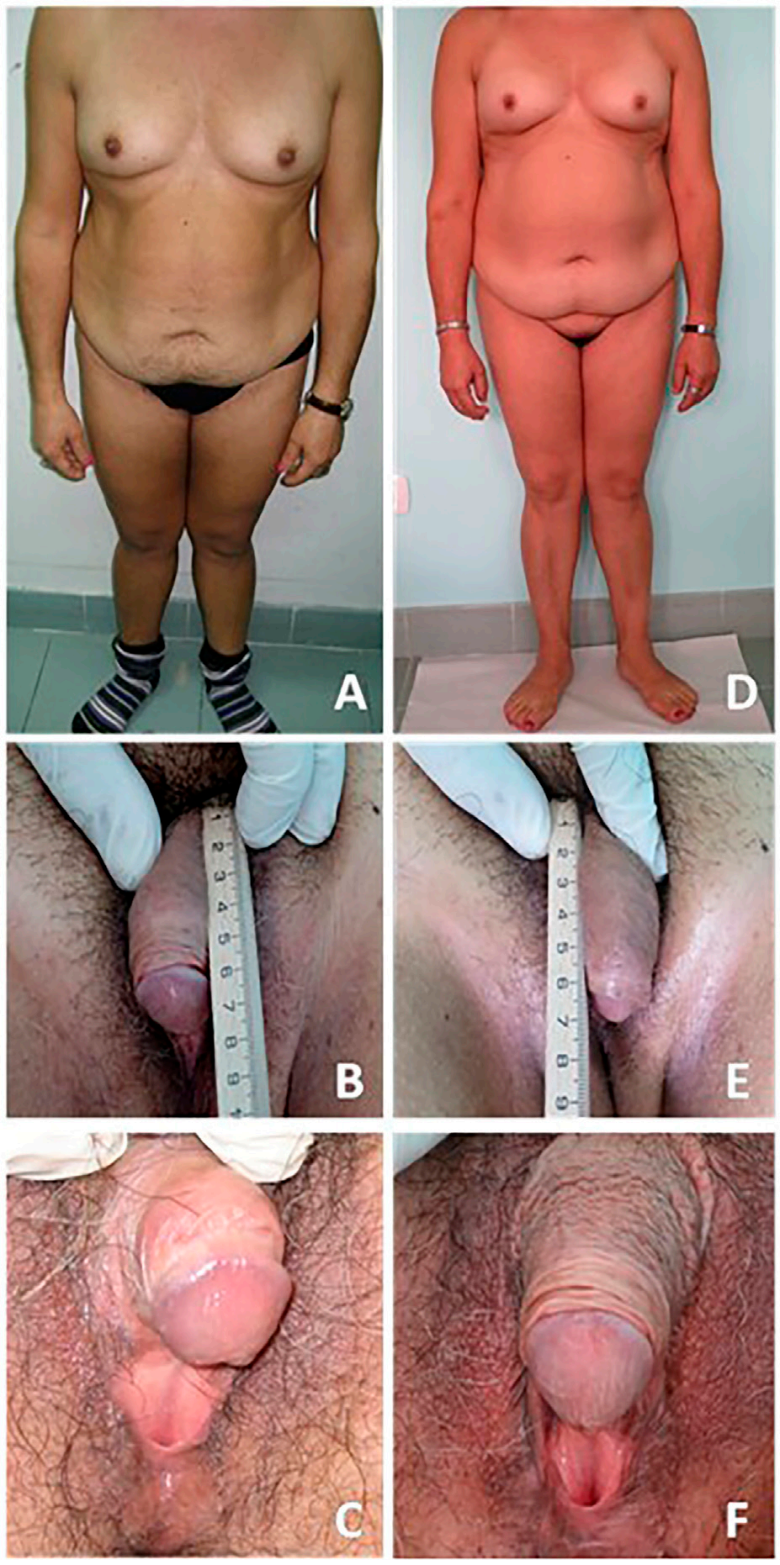
Patient bodily and genital characteristics. On the left side **(A–C)** are reported images related to the evaluation at diagnosis. On the right side **(D–F)** are reported images related to the last follow-up. A written informed consent was obtained by the patient in order to publish images and clinical data.

Table 1 shows clinical, biochemical, and hormonal relevant findings. Figure 1 shows patient’s body and genital characteristics, at diagnosis and at the last follow-up.

**TABLE 1:** Clinical, biochemical, and hormonal relevant findings at diagnosis and after 12 months of dual-release hydrocortisone treatment (5 years of GCs).

	Baseline	12 months of DRHC (5 years GCs)	Normal values
Clinical findings			
BMI (kg/m^2^)	28	24	<25
Waist circumference (cm)	97	85	<80
Ferriman-Gallwey (score)	25	12	<8
Biochemical findings			
Total cholesterol (mg/dl)	236	198	<190
HOMA-IR index (score)	2.7	0.73	<2.5
Hormonal findings			
DHEAS mcg/dl (nmol/L)	237 (6423)	36.6 (991.9)	18–244 (488–6612)
Δ4 -ANDROSTENEDIONE ng/ml (nmol/L)	15.4 (53.7)	1.2 (4.2)	1–4.5 (3.5–15.7)
17-OH-PROGESTERONE ng/ml (nmol/L)	510 (1545)	2.6 (7.9)	0.1–2.9 (0.3–8.8)
TESTOSTERONE ng/dl (nmol/L)	176 (6.1)	41 (1.4)	20–120 (0.7–4.2)
ACTH pg/ml (pmol/L)	116 (25.5)	8.4 (1.8)	10–52 (2.2–11.4)

DRHC, dual-release hydrocortisone; GCs, glucocorticoids; BMI, body mass index; HOMA-IR, homeostatic model assessment insulin resistance; DHEAS, dehydroepiandrosterone sulfate; ACTH, adrenocorticotropic hormone.

### Baseline psychosexual assessment

At initial interview, the patient referred, since childhood, a strong discrepancy between the assignment of a female gender and the self-perception of a male gender identity, with a clear discomfort in identifying herself as a female, mainly due to external genital ambiguity; nevertheless, the patient never self-presented as a male. The uncertainty in self-perception caused a severe impairment of patient psychological wellbeing, with uneasiness and disorientation, bringing to an almost complete isolation by affecting school insertion, work placement and social relationships. Notably, the occurrence of familial marginalization was associated with disregard of the severe psychological discomfort. Indeed, the patient reported a problematic relationship with her father, characterized by controlling behaviours and coercion; moreover, due to a patriarchal mentality and to a referred domestic abuse of the mother by the father, the mother was unable to effectively take care of the patient nor to face father coercive attitude. It is worth to note that mother died when the patient was 46 years old, few months after the disease diagnosis. The patient was therefore left alone with the great assignment to provide material, physical and medical support to her father. None of the patient’s relatives showed consideration of her physical and psychological discomfort, even forcing the patient to hide herself and avoid social connections. This familial environment and ensuing isolation prevented the patient to naturally compare herself with peers and to receive medical care. Indeed, the patient never sought for medical help, except when she referred to the hospital for abdominal pain at 46 years of age.

The psychosexual assessment of the patient included: 1) global psychological dimension, assessed by the self-report questionnaires Symptom Checklist-90-Revised (SCL-90-R) (Derogatis and Cleary, 1977; Derogatis, 1994) and Beck Depression Inventory II (BDI-II) (Beck et al., 1996); 2) gender identity dimension, assessed by the self-report questionnaires Gender Identity/Gender Dysphoria Questionnaire for Adolescents and Adults (GIDYQ-AA) (Deogracias et al., 2007), Utrecht Gender Dysphoria (UGDS) (Cohen-Kettenis and van Goozen, 1997; Schneider et al., 2016) together with Recalled Childhood Gender Identity (RCGI) Scale (Meyer-Bahlburg et al., 2006b; Zucker et al., 2006), the latter used as a retrospective tool to measure the recalled gender-typed behaviour and relative closeness to mother and father during childhood; 3) body image dimension, assessed by Body Uneasiness Test (BUT) (Cuzzolaro et al., 1999), including BUT-A (Global Severity Index) to measure the body image concern, and BUT-B (Positive Symptom Distress Index) to measure discomfort relative to specific body areas; 4) sexual orientation dimension, assessed by Coleman’s Clinical Interview (Coleman, 1987) and Klein Sex Orientation Grid (KSOG) (Klein et al., 1985; Klein, 1993); 5) sexual function dimension, assessed by Female Sexual Function Index (FSFI) (Rosen et al., 2000; Wiegel et al., 2005). Despite controversial opinions reported in scientific literature on its applicability to patients suffering from CAH (Meyer-Bahlburg and Dolezal, 2007), FSFI questionnaire, along with psychological interview, was considered the best tool to assess female sexual function quality.

Overall, the patient reported serious suffering related to psychological wellbeing, gender identity and role, and body image dimensions, as well as to sexual orientation and sexual function. The SCL-90-R revealed an overall psychological discomfort, with impairment in sub-domains referring to somatization, obsessive-compulsive behaviours, interpersonal sensitivity, depression, anxiety, hostility, paranoid ideation, psychoticism, and sleep disorders. Conversely, phobic anxiety domain was normal. The BDI-II questionnaire score revealed severe depressive symptoms; sub-scores suggested impaired mood, accompanied by somatic-affective symptoms and cognitive rigidity. The GIDYQ-AA and UGDS questionnaires suggested gender dysphoria: the patient experienced discomfort in perceiving herself in the female gender assigned at birth. The RCGI Scale revealed cross-gender behaviours experienced since infancy, and closeness with the mother. The BUT-A revealed a severe overall body image discomfort, with impairment in the sub-domains referring to body image concern, avoidance and depersonalization. The BUT-B revealed a severe body image discomfort related to specific areas, particularly, height, moustache, beard, breast, belly, and genitalia. Coleman’s Clinical Interview and KSOG revealed homosexual orientation, relative to the female gender assigned at birth, in all addressed dimensions. Lastly, FSFI score highlighted a severely compromised sexual function with impairment in desire, lubrication, arousal, orgasm, and general satisfaction, whereas pain was normal, probably due to the absence of vaginal penetrative sexual activity. Indeed, patient’s sexual activity was only based on autoerotic and purely external stimulation of genitalia; patient never experienced any vaginal penetrative sexuality neither with her own fingers nor with the employment of sex toys. In summary, at diagnosis, the patient showed an overall psychological discomfort associated with gender dysphoria, cross-gender behaviours, and a severe body image discomfort, self-perceiving as homosexual although sexual experience was uniquely autoerotic, partial and unsatisfying, therefore resulting in a severe sexual dysfunction.

Table 2 shows psychological, gender and sexual assessment at diagnosis and at the last follow-up.

**TABLE 2:** Psychological, sexual, and gender assessment at diagnosis and after 12 months of dual-release hydrocortisone treatment (5 years of GCs).

	Baseline	12 months of DRHC (5 years GCs)	Normal values or range scores
SCL-90-R			
Global severity index	2.06	0.43	<1
Somatization	2	0.58	<1
Obsessive-compulsive	2.20	0.50	<1
Interpersonal sensitivity	2.11	0.33	<1
Depression	3.15	0.77	<1
Anxiety	2.10	0.50	<1
Hostility	1	0.17	<1
Phobic anxiety	0.29	0	<1
Paranoid ideation	2.67	0.50	<1
Psychoticism	1.80	0.10	<1
Sleep disorders	3	0.9	<1
BDI-II			
Total score	36	2	≤13^a^
Somatic-affective	18	2	≤10
Cognitive	18	0	≤3
GIDYQ-AA			
Total score	2.18	4.4	>3
UGDS			
Total score	35	13	Range 12–60^b^
RCGI			
Factor 1	1.57	-	Range 0–5^c^
Factor 2	4.25	-	Range 0–5^d^
BUT-A			
Global severity index	3.32	0.26	≤1.2
Body image concern	4.78	0.67	<1
Avoidance	5	0	<1
Depersonalization	3	0	<1
BUT-B			
Positive symptom distress index	5	4.67	<1
FSFI			
Total score	16.9	26	>26.55
Desire	2.4	6	1.2–6
Lubrication	1.5	1.2	0–6
Arousal	3	4.8	0–6
Orgasm	2	4	0–6
Satisfaction	2	4	0.8–6
Pain	6	6	0–6

DRHC, dual-release hydrocortisone; GCs, glucocorticoids; SCL-90-R, Symptom Checklist-90-Revised; BDI-II, Beck Depression Inventory II; GIDYQ-AA, Gender Identity/Gender Dysphoria Questionnaire for Adolescents and Adults; UGDS, Utrecht Gender Dysphoria; RCGI, Recalled Childhood Gender Identity; BUT, Body Uneasiness Test; FSFI, Female Sexual Function Index (for all domains, lower within range scores are related to more severe impairment).

^a^ Range 14–19 mild, range 20–28 moderate, range 29–63 severe depression.

^b^ Higher within range scores are related to stronger gender dysphoria.

^c^ Factor 1 lower within range scores are related to greater recalled cross-gender behaviours.

^d^ Factor 2 lower within range scores are related to cross-gender identification.

### Follow-up

After 12 months of prednisone (7.5–15 mg/day), the patient was treated with conventional immediate release hydrocortisone (HC), maintained for 12 months at high doses of 60 mg/day (equivalent to prednisone 15 mg/day). HC doses were progressively reduced to 20 mg/day in the following 24 months. After 36 months of conventional HC treatment, the patient started a more physiologic GC, the dual-release HC (DRHC), at an equivalent dose of 20 mg/day, subsequently increased up to 25 mg/day during the following 12 months. At the age of 47 years, 18 months after the diagnosis, under HC treatment, a spontaneous menarche appeared. During GC treatment, the patient showed skin depigmentation, normalization in body weight and improvement in visceral adiposity and hirsutism*,* without changes in external genitalia*,* an improvement of hypercholesterolemia and the resolution of insulin resistance. Moreover, at follow-up, changes in patient’s familial and socio-cultural factors were registered; particularly, patient had started living alone and had interrupted social isolation.

At 5-year follow-up, after 12 months of DRHC treatment, and 5 years of GC treatment, a complete psychosexual assessment was repeated, except for the RCGI Scale, which refers to the infancy experience. The SCL-90-R revealed a global resolution in the overall psychological discomfort, with a complete normalization of all sub-domains. The BDI-II questionnaire highlighted a relevant improvement of mood, up to depressive symptoms resolution. The GIDYQ-AA and UGDS revealed gender dysphoria resolution. The BUT-A revealed a relevant improvement of global body image self-perception, with normalization of the sub-domains referring to body image concern, avoidance, and depersonalization. The BUT-B revealed disappearance of discomfort in the genital area, despite a persistent body image discomfort related to specific body areas, such as height, moustache, beard, breast, and belly. The Coleman’s Clinical Interview and KSOG did not highlight any modification, by confirming homosexual orientation relative to the female gender assigned at birth, however perceived without discomfort. Lastly, FSFI highlighted a relevant improvement in sexual function, and specifically in desire, arousal, orgasm, and general satisfaction with a persistent impairment of lubrication, which were all also referred to the experience of couple sexuality, consisting exclusively of external genitalia stimulation; nevertheless, the pain domain continued to be normal still considering the absence of any kind of sole/couple vaginal penetrative sexual activity. Hence, at follow-up, the patient showed an overall improvement of psychological and body image discomfort, with gender dysphoria resolution. Moreover, the patient continued to perceive herself as homosexual, and referred an improvement of sexual experience and function.

## Discussion

The current manuscript describes an unusual case of an Italian woman, homeborn in the 60s, with an extraordinarily late diagnosis of simple virilising classic form of CAH due to 21-OHD, determining a relevant impairment of both physical and psychosexual development; to the best of our knowledge, this is the first case including a complete psychosexual assessment at the diagnosis, with a detailed re-evaluation after 5 years of disease treatment.

At diagnosis, the patient of the current report displayed primary amenorrhea, height under target, overweight with visceral adiposity, hypercholesterolemia and insulin resistance, hirsutism with a typical male-pattern hair growth, external genital ambiguity, and a severe impairment in the entire series of psychological dimensions, particularly, severe depressive symptoms, together with body image discomfort. Moreover, the patient displayed gender dysphoria relative to the female gender assigned at birth and cross-gender behaviours, never resulted in an overt change to a male self-presentation, associated with homosexual orientation, and sexual dysfunction. A cluster of factors, including prenatal exposure to androgen excess, uncorrected external genital ambiguity, late diagnosis and treatment with consequent postnatal long-term severe uncontrolled androgen excess, together with familial and socio-cultural factors, seems to have contributed to the severe physical and psychosexual impairment.

In the classic forms of CAH due to 21-OHD, the pathophysiology as well as the eventual untimely diagnosis and treatment, because of the exposure of the patient initially to prenatal androgen excess, and thereafter to postnatal androgen excess up to hormonal control, may influence physical aspect, metabolic profile, gender assignment and identity, and psychosexual development, not only through structural effects on the developing brain, but also through body virilisation and subsequent impact on body image, psychological profile, and socialization (Dessens et al., 2005; Meyer-Bahlburg et al., 2016; Razzaghy-Azar et al., 2017; Speiser et al., 2018). Nowadays, the Endocrine Society Clinical Practice guidelines on clinical management of CAH due to 21-OHD recommend that the neonatal screening programs incorporate screening for CAH due to 21-OHD, because early diagnosis and treatment can prevent serious morbidity, mortality as well as impairment of quality of life (Speiser et al., 2018). In particular, in female patients with classic form of CAH, a correct female gender assignment, a prompt surgical treatment with external genital ambiguity correction, and an adequate disease treatment with control of androgen excess, permit to increase the probability to maintain a general wellbeing, to perceive a female gender identity, and to best manage the sexual dysfunctions, especially related to the dyspareunia (Speiser et al., 2018).

In patients with classic form of CAH due to 21-OHD, metabolic complications have been reported in 40.4%–80.7% of cases, displaying greater rates compared with the prevalence of 19.2%–41% of cases reported in the United States general population, considering the most recent and large longitudinal study (Torky et al., 2021). Particularly, in CAH patients compared to the general population, reported prevalences were: 49.1% and 34.5% for visceral obesity, 63.2% and 19.2% for arterial hypertension, 80.7% and 41% for insulin resistance, 45.6% and 35.5% for fasting hyperglycemia, and 40.4% and 29.5% for low HDL cholesterol levels (Torky et al., 2021). Both the cortisol deficiency, with the subsequent androgen excess (Zhang et al., 2010), and the GC treatment-related hypercortisolism, mimicking iatrogenic hypercortisolism (Tamhane et al., 2018), may predispose patients to the development of metabolic complications, since childhood to adulthood (Speiser et al., 2018). In the patient of the current report, metabolic complications including overweight, visceral adiposity, hypercholesterolemia, and insulin resistance, highlighted by clinical evaluation and biochemical assessment, might have been determined by the occurrence of late diagnosis and absence of a prompt treatment, with persistent postnatal exposure to androgen excess. Noteworthy, the patient revealed body image discomfort related to visceral adiposity, as demonstrated by body image dimension assessment performed using BUT questionnaire, potentially contributing to the psychological discomfort (Marano et al., 2007). In particular, derision for overweight and shame and blame feelings about an overweight body size, highlighted by clinical interview, might have been hypothesized to have further contributed to the severe body image concern (Schwartz and Brownell, 2004) and consequently to the psychological discomfort of the patient of the current report.

In female patients with classic form of CAH, mental health problems, and particularly depression, have been reported in up to 40.2% of cases in a large case series, displaying slightly greater rate compared with the prevalence of 32.5% of cases reported in the United Kingdom general population, considering a recent large retrospective study (Jenkins-Jones et al., 2018). The cortisol deficiency with the higher risk of potentially fatal electrolyte crises (Speiser et al., 2018), as well as the prenatal (Kuhnle et al., 1995; Zucker et al., 1996; Hines et al., 2004; Dessens et al., 2005; Meyer-Bahlburg et al., 2006a; Meyer-Bahlburg et al., 2008; Frisén et al., 2009; Hines et al., 2015; Meyer-Bahlburg et al., 2016; Razzaghy-Azar et al., 2017; Spencer et al., 2017; Speiser et al., 2018; Daae et al., 2020) and postnatal (Jenkins-Jones et al., 2018; Speiser et al., 2018) exposure to androgen excess with consequent effects on body and brain, may be individualized as key risk factors in developing mental health problems in female patients with classic form of CAH, with significant challenges not only for affected individuals but also for their parents (Speiser et al., 2018). In the patient of the current report, the psychological discomfort, and particularly the severe depressive symptoms, highlighted by both clinical interview and global psychological assessment, performed using SCL-90-R and BDI-II questionnaires, might have been determined by all key risk factors reported in literature, such as cortisol deficiency, prenatal and postnatal exposure to androgen excess, and might have been exacerbated by a combination of additional factors specifically characterizing the patient of the current report, and leading to late diagnosis and absence of an adequate treatment. Homebirth in the 60s with the only support of a midwife was the first factor not allowing a prompt diagnosis; familial and socio-cultural factors leading to social isolation, along with the presence of a subtle clinical picture of adrenal insufficiency, not characterized by overt signs and symptoms, which frequently guide the diagnosis, determined avoiding of medical care until the age of 46 years. This delayed diagnosis prevented the external genital ambiguity correction and the appropriate GC treatment, with persistent postnatal exposure to androgen excess, leading to severe body image discomfort related to height, visceral adiposity, diffuse hirsutism, inadequate breast and genitalia, as demonstrated by body image dimension assessment, performed using BUT questionnaire. Lastly, familial coercion and social deprivation might be hypothesized to have further and independently contributed to the psychological discomfort and depressive symptoms.

In female patients with classic form of CAH, gender-related problems have been reported in up to 3% of cases, displaying a greater rate compared with the prevalence of 0.002%–0.003% reported in assigned females at birth (Fisher et al., 2018), specifically gender dysphoria and cross-gender behaviours, including intense physical energy expenditure and preference for traditionally masculine activities (Hines, 1982). Nevertheless, the prevalence of gender-related problems in assigned females at birth could be underestimated due to fluctuations in gender-variant identities (Zucker, 2017); indeed, some studies have reported in female general population a prevalence of 0.6%–0.8% of “gender incongruent” feelings (Kuyper and Wijsen, 2014; Van Caenegem et al., 2015). Noteworthy, despite the underestimation in assigned females at birth, prevalence of gender-related problems remains higher in CAH female patients. Prenatal exposure to androgen excess in female patients with classic form of CAH has been individualized as the key risk factor in developing their gender-related problems, acting on brain development, and exerting masculinizing effects, despite timely treatment and irrespective of socio-cultural background (Seneviratne et al., 2021). Indeed, both neuroanatomical and functional differences have been described, in humans, in relation to gender identity variation in some including the bed nucleus of the stria terminalis in hypothalamus and the third interstitial nucleus of the anterior hypothalamus; the development of both hypothalamic areas has been demonstrated to be dependent on prenatal androgens exposure, in animal models (Fisher et al., 2018; Korpaisarn and Safer, 2019). However, hormonal control, as well as familial and socio-cultural factors may have a further possible impact (Razzaghy-Azar et al., 2017). In the patient of the current report, the gender dysphoria and the cross-gender behaviours, highlighted by both clinical interview and gender identity dimension assessment, performed using GIDYQ-AA, UGDS and RCGI questionnaires, might have been determined by the key risk factors reported in literature (Razzaghy-Azar et al., 2017; Seneviratne et al., 2021), such as prenatal exposure to androgen excess, hormonal control, as well as familial and socio-cultural factors, and might be hypothesized to have been exacerbated by a combination of additional factors specifically characterizing the patient of the current report, particularly, the long-term uncorrected genital ambiguity, along with persistent exposure to androgen excess since infancy throughout adulthood, also determining body masculinization and generating severe body image discomfort related to specific areas including mainly genitalia, but also diffuse hirsutism and inadequate breast, as demonstrated by body image dimension assessment, performed using BUT questionnaire. Lastly, familial constraints, specifically, the perceived mismatch between the presence of ambiguous genitalia and the requirement to live, wear and behave as a female, in order to indulge parents’ expectations, highlighted by clinical interview, might have been hypothesized to have further contributed to the development of gender dysphoria.

In female patients with classic and non-classic forms of CAH, non-heterosexuality is relatively frequent; indeed, bisexuality and homosexuality have been reported in 3.4%–37% and 3%–20% of cases, respectively (Daae et al., 2020), displaying greater rates compared with the prevalence of 7.2% and 2.1% of cases, respectively, reported across the general population of 28 nations, considering a recent large cross-sectional study (Rahman et al., 2020). Nevertheless, these prevalences in the general population could be underestimated as suggested by a recent large study demonstrating, in women, a prevalence but a prevalence of predominantly, not exclusively, heterosexual women of 66.2% (Rahman et al., 2020). Noteworthy, despite the underestimation in the general population, the prevalence of pure non-heterosexuality remains higher in CAH female patients. Prenatal exposure to androgen excess in female patients with classic and non-classic forms of CAH has been individualized as a key risk factor in developing bisexuality and homosexuality, acting on brain development (Daae et al., 2020). Indeed, both differences have been described, in humans and animal models, in relation to sexual orientation, in particular in the third interstitial nucleus of the anterior hypothalamus, whose development has been demonstrated to be dependent on prenatal androgens exposure in animal models (Fisher et al., 2018). Nevertheless, the impact of prolonged exposure to androgen excess since infancy and throughout adulthood should not be underestimated. Some evidence suggests that a higher frequency of long-term homosexual relationships is observed in female patients with simple virilising classic form of CAH, compared with salt wasting classic form of CAH, suggesting that a delayed treatment, typical of simple virilising forms, may have a role in the increased frequency of homosexuality, by determining a longer term androgenization (Daae et al., 2020). Conversely, different evidence suggests that sexual orientation appears to be related to CAH severity, since the most severe genotypes, such as those associated with salt wasting forms, reported a higher rate of bisexuality and homosexuality, compared with lower rates in the less severe genotypes, such as those associated with simple virilising classic forms of CAH and non-classic forms of CAH (Frisén et al., 2009; Daae et al., 2020)*.* In the patient of the current report, the homosexual orientation highlighted by both clinical interview and sexual orientation dimension assessment, performed using Coleman’s Clinical Interview and KSOG questionnaire, might have been determined by the key risk factors reported in literature (Daae et al., 2020), such as prenatal and postnatal exposure to androgen excess, and might have been exacerbated by a combination of additional factors specifically characterizing the patient of the current report, including the long duration of uncontrolled disease, and the masculine physical appearance, which could be hypothesized to have a role on sexual interest. Lastly, the presence of the coercive figure of the father (Roberts et al., 2013), highlighted by clinical interview, might have been hypothesized to have further contributed to the homosexuality, due to discomfort in interacting with the males. Both clinical interview and sexual orientation dimension assessment revealed a severe discomfort for the “perceived” sexual orientation with homosexual positioning. This discomfort could be hypothesized to be related to gender identity disorientation as well as to body image concern, in particular referred to genitalia, as demonstrated by body image dimension assessment, performed using BUT questionnaire. Beyond the different hypotheses linking the homosexuality to the clinical condition, the possibility that the homosexuality of the patient might be independent from the presence of CAH, representing as an intrinsic sexual orientation of the patient, cannot be ruled out.

In female patients with classic form of CAH, sexual dysfunctions have been described in 71% of cases, compared with the prevalence of 37% reported in healthy women, not affected by CAH (Dwiggins et al., 2020). The major limitation in sexual function appeared to be dyspareunia, although impairment of desire, lubrication, arousal, orgasm, and general satisfaction, have also been reported (Gastaud et al., 2007; Dwiggins et al., 2020). Nevertheless, the complete lack of heterosexual intercourse with vaginal penetration (37%) or of sexual activity (8.6%) have also been documented (Gastaud et al., 2007). Some evidence suggests that a worse sexual function with major sexual concerns and fewer sexual relationships with partners are observed in female patients with salt wasting classic form of CAH compared with female patients with simple virilising classic form of CAH (Wisniewski et al., 2004). The masculinization of external genitalia, and particularly the presence of smaller vaginal orifice and larger clitoris (Dwiggins et al., 2020), as well as the extensive reconstructive surgery, with consequent reduced sensitivity and orgasmic capacity, and reduced oestrogen levels due to impaired ovarian function induced by androgen excess, have been individualized as key risk factors in developing sexual dysfunction in CAH (Gastaud et al., 2007); the same factors might also be hypothesized to explain the worse outcome in salt wasting classic form of CAH, compared with simple virilising classic form of CAH, indirectly due to a higher degree of cortisol deficiency determining greater masculinization, and the need for more extensive surgery (Wisniewski et al., 2004). In the patient of the current report, the sexual dysfunction highlighted by both clinical interview and sexual function dimension, assessed by FSFI questionnaire, might have been determined by one of the two key risk factors reported in literature (Gastaud et al., 2007), such as the high degree of masculinization of external genitalia, which persisted, since infancy to adulthood, due to the familial and socio-cultural factors preventing surgical correction. Moreover, sexual dysfunction might have been exacerbated by a combination of additional factors specifically characterizing the patient of the current report, including body masculinization generating severe body image discomfort, mainly related to genitalia (Gastaud et al., 2007), as demonstrated by body image dimension assessment performed using BUT questionnaire, and familial and socio-cultural factors determining the patient’s social marginalisation and extremely low self-esteem, with deprivation of affective and sexual relationships. Both clinical interview and sexual function dimension assessment revealed a heavily compromised sexual function with severe impairment in desire, lubrication, arousal, orgasm, and general satisfaction. Notably, pain was normal, but this was probably due to the absence of vaginal penetrative sexual activity. Indeed, the patient reported by clinical interview an intense attitude to autoerotic experiences, with purely external stimulation of genitalia, and that never experienced any vaginal penetrative sexuality neither with fingers nor with the employment of sex toys.

At follow-up, the patient of the current report displayed a spontaneous menarche appearance, an improvement in hirsutism, normalization in body weight, an improvement in visceral adiposity and hypercholesterolemia, and a resolution of insulin resistance, accompanied by physical aspect changes to more feminine characteristics. Notably, the patient showed resolution of psychological discomfort and remission of severe depressive symptoms as well as gender dysphoria. The patient felt a relevant overall improvement in body image self-perception, with no more discomfort reported in the genital area, associated with confirmation of homosexual orientation, and with a relevant improvement in sexual dysfunction. A cluster of factors, including diagnosis and treatment with consequent androgen excess control and related physical aspect changes, together with familial and socio-cultural factors changes, particularly, living alone and distancing from father controlling behaviours and coercive attitudes, with the achievement of a higher independence and self-esteem, and the interruption of social isolation, seems to have contributed to the psychosexual improvement of the study patient.

Homosexual orientation was the only psychosexual trait apparently unchanged after diagnosis and GC treatment, although it was gradually accepted and perceived without discomfort; the disappearance of the discomfort is not surprising considering that homosexual orientation discomfort was hypothesized to be related to gender identity disorientation, body image concern and familial and socio-cultural factors. Indeed, after diagnosis and treatment, the patient felt more conscious concerning gender identity, and fully recognized herself as a woman, gradually experiencing a change of gender identity accompanied by a shift from trans-gender to cis-gender behaviours, and started to experience affective relationships and couple homosexual activity, characterized by external genitalia stimulation with absence of any kind of sole/couple vaginal penetrative sexual activity, probably due to the improvement of the global body image self-perception.

Few additional cases of female patients with CAH due to 21-OHD, receiving an extremely delayed diagnosis, have been published (Lewin et al., 1980; Norris et al., 1996; Falhammar and Thoren, 2005; Khan et al., 2009). Similarly to the current case, previous cases highlighted that a delayed diagnosis may be due to various factors such as the absence of a typical clinical syndrome and laboratory evidence suggesting CAH or adrenal insufficiency (Falhammar and Thoren, 2005), the lack of awareness and reluctance in seeking medical care (Khan et al., 2009) with incidental diagnosis for the coincidence of abdominal masses requiring medical assistance and investigation (Lewin et al., 1980; Norris et al., 1996; Falhammar and Thoren, 2005; Khan et al., 2009), the presence of familial marginalization and social isolation, specifically in some countries, where many women do not routinely see a gynaecologist at any stage as an adult (Khan et al., 2009). However, this is the first case including a complete psychosexual assessment at diagnosis with a detailed description of psychosexual changes following treatment during a long-term follow-up period. All cases reported in the literature (Lewin et al., 1980; Norris et al., 1996; Falhammar and Thoren, 2005; Khan et al., 2009) strongly suggested that CAH diagnosis should be considered in any patient presenting with an adrenal mass, independently from the presence of clinical or laboratory signs of virilisation, and specifically at any age, including adulthood. Particularly, a systematic review and metanalysis reported that approximately 10% of patients with adrenal incidentaloma were carriers of a CYP21A2 mutation, highlighting the need for suspicion of CAH in patients with adrenal incidentalomas (Falhammar and Torpy, 2016).

In conclusion, the current case report highlights that, despite the evidence of external genital ambiguity since birth, the patient, homeborn in Italy during the 60s, received, at the age of 46 years, an extraordinarily late diagnosis of simple virilising classic form of CAH due to 21-OHD. This delayed diagnosis and consequent delayed treatment were mainly due to familial and socio-cultural factors, including disregard of patient’s clinical condition since birth throughout life, and deprivation of medical care, due initially to familial conditioning and thereafter to patient’s reluctance and social isolation. A minor role in delaying diagnosis might have been played by the presence of a subtle form of adrenal insufficiency, with no overt clinical syndrome, particularly salt wasting episodes. This relevant diagnostic delay determined severe long-term consequences, in terms of physical aspect, general psychological wellbeing, gender identity, body image self-perception, and sexual orientation and function; all of these long-term consequences successfully reverted following diagnosis and GC treatment, except for patient’s homosexual orientation, which persisted, although without discomfort. Therefore, the complete psychosexual assessment at diagnosis and the detailed description of psychosexual changes following treatment during a long-term follow-up period, firstly described in the current case report, suggest the relevant and deleterious role of delayed diagnosis and treatment on psychosexual outcomes in CAH female patients.

## Data Availability

The raw data supporting the conclusion of this article will be made available by the authors, without undue reservation.

## References

[B1] Beck A. T. Steer R. A. Ball R. Ranieri W. (1996). Comparison of Beck depression inventories -IA and -II in psychiatric outpatients. J. Pers. Assess. 67 (3), 588–597. 10.1207/s15327752jpa6703_13 8991972

[B2] Claahsen-van der Grinten H. L. Speiser P. W. Ahmed S. F. Arlt W. Auchus R. J. Falhammar H. (2022). Congenital adrenal hyperplasia-current insights in pathophysiology, diagnostics, and management. Endocr. Rev. 43 (1), 91–159. 10.1210/endrev/bnab016 33961029 PMC8755999

[B3] Clayton P. E. Miller W. L. Oberfield S. E. Ritzen E. M. Sippell W. G. Speiser P. W. (2002). Consensus statement on 21-hydroxylase deficiency from the European society for paediatric Endocrinology and the lawson wilkins pediatric endocrine society. Horm. Res. 58, 188–195. 10.1159/000065490 12324718

[B4] Cohen-Kettenis P. T. van Goozen S. H. (1997). Sex reassignment of adolescent transsexuals: a follow-up study. J. Am. Acad. Child. Adolesc. Psychiatry 36 (2), 263–271. 10.1097/00004583-199702000-00017 9031580

[B5] Coleman E. (1987). Assessment of sexual orientation. J. Homosex. 14 (1/2), 9–24. 10.1300/J082v14n01_02 3655356

[B6] Cuzzolaro M. Vetrone G. Marano G. Battacchi M. W. (1999). BUT body uneasiness test: a new attitudinal body image scale. Psichiatr. dell’infanzia dell’adolescenza 66, 417–428.

[B7] Daae E. Feragen K. B. Waehre A. Nermoen I. Falhammar H. (2020). Sexual orientation in individuals with congenital adrenal hyperplasia: A systematic review. Front. Behav. Neurosci. 14, 38. 10.3389/fnbeh.2020.00038 32231525 PMC7082355

[B8] Deogracias J. J. Johnson L. L. Meyer-Bahlburg H. F. Kessler S. J. Schober J. M. Zucker K. J. (2007). The gender identity/gender dysphoria questionnaire for adolescents and adults. J. Sex. Res. 44 (4), 370–379. 10.1080/00224490701586730 18321016

[B9] Derogatis L. R. Cleary P. A. (1977). Confirmation of the dimensional structure of the scl-90: A study in construct validation. J. Clin. Psychol. 33 (4), 981–989. 10.1002/1097-4679(197710)33:4<981:aid-jclp2270330412>3.0.co;2-0

[B10] Derogatis L. R. (1994). Symptom checklist-90-R: Administration, scoring and procedures manual. 3rd ed. Minneapolis: National Computer Systems.

[B11] Dessens A. B. Slijper F. M. Drop S. L. (2005). Gender dysphoria and gender change in chromosomal females with congenital adrenal hyperplasia. Arch. Sex. Behav. 34, 389–397. 10.1007/s10508-005-4338-5 16010462

[B12] Dwiggins M. Brookner B. Fowler K. Veeraraghavan P. Gomez-Lobo V. Merke D. P. (2020). Multidimensional aspects of female sexual function in congenital adrenal hyperplasia: A case-control study. J. Endocr. Soc. 4 (11), bvaa131. 10.1210/jendso/bvaa131 34485799 PMC7594652

[B13] Falhammar H. Thoren M. (2005). Thorén M an 88-year-old woman diagnosed with adrenal tumor and congenital adrenal hyperplasia: Connection or coincidence? J. Endocrinol. Invest. 28 (5), 449–453. 10.1007/bf03347226 16075929

[B14] Falhammar H. Torpy D. J. (2016). Congenital adrenal hyperplasia due to 21-hydroxylase deficiency presenting as adrenal incidentaloma: a systematic review and meta-analysis. Endocr. Pract. 22 (6), 736–752. 10.4158/EP151085.RA 26919651

[B15] Fisher A. D. Ristori J. Morelli G. Maggi M. (2018). The molecular mechanisms of sexual orientation and gender identity. Mol. Cell. Endocrinol. 467, 3–13. 10.1016/j.mce.2017.08.008 28847741

[B16] Frisén L. Nordenström A. Falhammar H. Filipsson H. Holmdahl G. Janson P. O. (2009). Gender role behavior, sexuality, and psychosocial adaptation in women with congenital adrenal hyperplasia due to CYP21A2 deficiency. J. Clin. Endocrinol. Metab. 94, 3432–3439. 10.1210/jc.2009-0636 19567521

[B17] Gastaud F. Bouvattier C. Duranteau L. Brauner R. Thibaud E. Kutten F. (2007). Impaired sexual and reproductive outcomes in women with classical forms of congenital adrenal hyperplasia. J. Clin. Endocrinol. Metab. 92, 1391–1396. 10.1210/jc.2006-1757 17284631

[B18] Gidlöf S. Falhammar H. Thilén A. von Döbeln U. Ritzén M. Wedell A. (2013). One hundred years of congenital adrenal hyperplasia in Sweden: a retrospective, population-based cohort study. Lancet. Diabetes Endocrinol. 1 (1), 35–42. 10.1016/S2213-8587(13)70007-X 24622265

[B19] Hines M. Brook C. Conway G. S. (2004). Androgen and psychosexual development: Core gender identity, sexual orientation and recalled childhood gender role behavior in women and men with congenital adrenal hyperplasia (CAH). J. Sex. Res. 41 (1), 75–81. 10.1080/00224490409552215 15216426

[B20] Hines M. Constantinescu M. Spencer D. (2015). Early androgen exposure and human gender development. Biol. Sex. Differ. 6, 3. eCollection 2015. 10.1186/s13293-015-0022-1 25745554 PMC4350266

[B21] Hines M. (1982). Prenatal gonadal hormones and sex differences in human behavior. Psychol. Bull. 92 (1), 56–80. 10.1037/0033-2909.92.1.56 7134329

[B22] Jenkins-Jones S. Parviainen L. Porter J. Withe M. Whitaker M. J. Holden S. E. (2018). Poor compliance and increased mortality, depression and healthcare costs in patients with congenital adrenal hyperplasia. Eur. J. Endocrinol. 178 (4), 309–320. 10.1530/EJE-17-0895 29371334

[B23] Khan A. H. Aban M. Hassan R. U. Hul Haq N. Raza J. Jabbar A. (2009). Classic virilizing congenital adrenal hyperplasia presenting late: Case series from Pakistan. J. Pak. Med. Assoc. 59 (9), 643–646. 19750867

[B24] Klein F. Sepekoff B. Wolf T. J. (1985). Sexual orientation: A multivariable dynamic process. J. Homosex. 11 (1/2), 35–49. 10.1300/J082v11n01_04 4056393

[B25] Klein F. (1993). The bisexual option. Binghamton, NY: Haworth Press.

[B26] Korpaisarn S. Safer J. D. (2019). Etiology of gender identity. Endocrinol. Metab. Clin. North Am. 48 (2), 323–329. 10.1016/j.ecl.2019.01.002 31027542

[B27] Kuhnle U. Bullinger M. Schwarz H. P. (1995). The quality of life in adult female patients with congenital adrenal hyperplasia: a comprehensive study of the impact of genital malformations and chronic disease on female patients life. Eur. J. Pediatr. 154 (9), 708–716. 10.1007/BF02276713 8582420

[B28] Kuyper L. Wijsen C. (2014). Gender identities and gender dysphoria in the Netherlands. Arch. Sex. Behav. 43, 377–385. 10.1007/s10508-013-0140-y 23857516

[B29] Lee P. A. Nordenström A. Houk C. P. Ahmed S. F. Auchus R. Baratz A. Global DSD Update Consortium (2016). Global disorders of sex development update since 2006: Perceptions, approach and care. Horm. Res. Paediatr. 85, 158–180. 10.1159/000442975 26820577

[B30] Lewin A. Kraiem Z. Kahana L. Sheinfeld M. Abramovici H. Lurie M. (1980). Congenital adrenal hyperplasia diagnosed in a middle-aged woman. Isr. J. Med. Sci. 16 (8), 607–609. 6968308

[B31] Marano G. Cuzzolaro M. Vetrone G. Garfinkel P. E. Temperilli F. Spera G. (2007). Validating the body uneasiness test (BUT) in obese patients. Eat. Weight Disord. 12 (2), 70–82. 10.1007/BF03327581 17615491

[B32] Meyer-Bahlburg H. Dolezal C. (2007). The female sexual function index: a methodological critique and suggestions for improvement. J. Sex. Marital Ther. 33, 217–224. 10.1080/00926230701267852 17454519

[B33] Meyer-Bahlburg H. F. Dolezal C. Baker S. W. Ehrhardt A. A. New M. I. (2006). Gender development in women with congenital adrenal hyperplasia as a function of disorder severity. Arch. Sex. Behav. 35 (6), 667–684. Epub 2006 Aug 11. 10.1007/s10508-006-9068-9 16902816

[B34] Meyer-Bahlburg H.F. Dolezal C. Zucker K. J. Kessler S. J. Schober J. M. New M. I. (2006). The recalled childhood gender questionnaire-revised: A psychometric analysis in a sample of women with congenital adrenal hyperplasia. J. Sex. Res. 43 (4), 364–367. 10.1080/00224490609552335 17599257

[B35] Meyer-Bahlburg H. F. Dolezal C. Baker S. W. New M. I. (2008). Sexual orientation in women with classical or non-classical congenital adrenal hyperplasia as a function of degree of prenatal androgen excess. Arch. Sex. Behav. 37 (1), 85–99. 10.1007/s10508-007-9265-1 18157628

[B36] Meyer-Bahlburg H. F. BaratzDalke K. Berenbaum S. A. Cohen-Kettenis P. T. Hines M. Schober J. M. (2016). Gender assignment, reassignment and outcome in disorders of sex development: Update of the 2005 consensus conference. Horm. Res. Paediatr. 85, 112–118. 10.1159/000442386 26727471

[B37] Murphy C. Allen L. Jamieson M. A. (2011). Ambiguous genitalia in the newborn: an overview and teaching tool. J. Pediatr. Adolesc. Gynecol. 24, 236–250. 10.1016/j.jpag.2011.02.004 21872773

[B38] Norris A. M. O'Driscoll J. B. Mamtora H. (1996). Macronodular congenital adrenal hyperplasia in an adult with female pseudohermaphroditism. Eur. Radiol. 6 (4), 470–472. 10.1007/BF00182473 8798026

[B39] Rahman Q. Xu Y. Lippa R. A. Vasey P. L. (2020). Prevalence of sexual orientation across 28 nations and its association with gender equality, economic development, and individualism. Arch. Sex. Behav. 49 (2), 595–606. 10.1007/s10508-019-01590-0 31797225 PMC7031179

[B40] Razzaghy-Azar M. Karimi S. Shirazi E. (2017). Gender identity in patients with congenital adrenal hyperplasia. Int. J. Endocrinol. Metab. 15 (3), e12537. 10.5812/ijem.12537 29201068 PMC5701969

[B41] Roberts A. L. Glymour M. M. Koenen K. C. (2013). Does maltreatment in childhood affect sexual orientation in adulthood? Arch. Sex. Behav. 42, 161–171. 10.1007/s10508-012-0021-9 22976519 PMC3535560

[B42] Rosen R. Brown C. Heiman J. Leiblum S. Meston C. Shabsigh R. (2000). The female sexual function index (FSFI): a multidimensional self-report instrument for the assessment of female sexual function. J. Sex. Marital Ther. 26 (2), 191–208. 10.1080/009262300278597 10782451

[B43] Schernthaner-Reiter M. H. Baumgartner-Parzer S. Egarter H. C. Krebs M. Kautzky-Willer A. Kirchheiner K. (2019). Influence of genotype and hyperandrogenism on sexual function in women with congenital adrenal hyperplasia. J. Sex. Med. S1743-6095 (19), 1529–1540. 10.1016/j.jsxm.2019.07.009 31447379

[B44] Schneider C. Cerwenka S. Nieder T. O. Briken P. Cohen-Kettenis P. T. De Cuypere G. (2016). Measuring gender dysphoria: A multicenter examination and comparison of the Utrecht gender dysphoria scale and the gender identity/gender dysphoria questionnaire for adolescents and adults. Arch. Sex. Behav. 45 (3), 551–558. 10.1007/s10508-016-0702-x 26883025

[B45] Schwartz M. B. Brownell K. D. (2004). Obesity and body image. Body Image 1 (1), 43–56. 10.1016/S1740-1445(03)00007-X 18089140

[B46] Seneviratne S. N. Jayarajah U. Gunawardana S. Samarasinghe M. de Silva S. (2021). Gender-role behaviour and gender identity in girls with classical congenital adrenal hyperplasia. BMC Pediatr. 21 (1), 262. 10.1186/s12887-021-02742-9 34090382 PMC8178869

[B47] Speiser P. W. Arlt W. Auchus R. J. Baskin L. S. Conway G. S. Merke D. P. (2018). Congenital adrenal hyperplasia due to steroid 21-hydroxylase deficiency: An endocrine society clinical Practice guideline. J. Clin. Endocrinol. Metab. 103 (11), 4043–4088. 10.1210/jc.2018-01865 30272171 PMC6456929

[B48] Spencer D. Pasterski V. Neufeld S. Glover V. O'Connor T. G. Hindmarsh P. C. (2017). Prenatal androgen exposure and children's aggressive behavior and activity level. Horm. Behav. 96, 156–165. Epub 2017 Nov 1. 10.1016/j.yhbeh.2017.09.012 28939371 PMC5722694

[B49] Tamhane S. Rodriguez-Gutierrez R. Iqbal A. M. Prokop L. J. Bancos I. Speiser P. W. (2018). Cardiovascular and metabolic outcomes in congenital adrenal hyperplasia: A systematic review and meta-analysis. J. Clin. Endocrinol. Metab. 103 (11), 4097–4103. 10.1210/jc.2018-01862 30272185

[B50] Torky A. Sinaii N. Jha S. Desai J. El-Maouche D. Mallappa A. (2021). Cardiovascular disease risk factors and metabolic morbidity in a longitudinal study of congenital adrenal hyperplasia. J. Clin. Endocrinol. Metab. 106 (12), e5247–e5257. 10.1210/clinem/dgab133 33677504 PMC8864751

[B51] Van Caenegem E. Wierckx K. Elaut E. Buysse A. Dewaele A. Van Nieuwerburgh F. (2015). Prevalence of gender nonconformity in Flanders, Belgium. Arch. Sex. Behav. 44, 1281–1287. 10.1007/s10508-014-0452-6 25588709

[B52] Wiegel M. Meston C. Rosen R. (2005). The female sexual function index (FSFI): Cross-validation and development of clinical cutoff scores. J. Sex. Marital Ther. 31 (1), 1–20. 10.1080/00926230590475206 15841702

[B53] Wisniewski A. B. Migeon C. J. Malouf M. A. Gearhart J. P. (2004). Psychosexual outcome in women affected by congenital adrenal hyperplasia due to 21-hydroxylase deficiency. J. Urol. 171, 2497–2501. 10.1097/01.ju.0000125269.91938.f7 15126884

[B54] Zhang H. J. Yang J. Zhang M. N. Liu C. Q. Xu M. Li X. J. (2010). Metabolic disorders in newly diagnosed young adult female patients with simple virilizing 21-hydroxylase deficiency. Endocrine 38 (2), 260–265. 10.1007/s12020-010-9382-9 20978868

[B55] Zucker K. J. Bradley S. J. Oliver G. Blake J. Fleming S. Hood J. (1996). Psychosexual development of women with congenital adrenal hyperplasia. Horm. Behav. 30 (4), 300–318. 10.1006/hbeh.1996.0038 9047259

[B56] Zucker K. J. Mitchell J. N. Bradley S. J. Tkachuk J. Cantor J. M. Allin S. M. (2006). The recalled childhood gender identity/gender role questionnaire: Psychometric properties. Sex. Roles 54 (7-8), 469–483. 10.1007/s11199-006-9019-x

[B57] Zucker K. J. (2017). Epidemiology of gender dysphoria and transgender identity. Sex. Health 14 (5), 404–411. 10.1071/SH17067 28838353

